# Association between high DHEAS in 7-year-old girls and metabolic features 4 years after menarche

**DOI:** 10.1210/jendso/bvaf211

**Published:** 2026-01-14

**Authors:** Ana Pereira, Gordon B Cutler, German Iñiguez, Verónica Mericq

**Affiliations:** Institute of Nutrition and Food Technology (INTA), University of Chile, Santiago 3510000, Chile; Consultancy LLC, Deltaville, VA 23043, USA; Institute of Maternal and Child Research (IDIMI), Faculty of Medicine, University of Chile, Santiago 8360160, Chile; Institute of Maternal and Child Research (IDIMI), Faculty of Medicine, University of Chile, Santiago 8360160, Chile

**Keywords:** adrenarche, metabolism, obesity, metabolic syndrome, 11 oxo steroids, DHEAS

## Abstract

**Background:**

Premature adrenarche is linked to increased adiposity, enhanced adrenal 11-oxygenated C19 androgen production, and adverse metabolic profiles in childhood. However, it remains unclear whether early elevations in dehydroepiandrosterone sulfate (DHEAS) predict metabolic dysfunction in adolescence or young adulthood.

**Methods:**

This longitudinal study included girls from the Growth and Obesity Chilean Cohort Study, all with normal birth weight. DHEAS was measured at ∼7 years (n = 504, 68% follow-up), and participants were reevaluated 4 years after menarche (4YPM, ∼16 years). Girls were classified as having high DHEAS (HD; >75th percentile) or normal DHEAS (ND; ≤75th percentile). Anthropometric, metabolic, and hormonal parameters, including 11-oxygenated C19 steroids, were compared between groups.

**Results:**

At 4YPM, HD girls (n = 96) showed higher body mass index SD score (*P* < .01), waist-to-height and waist-to-hip ratios (*P* < .05), and 47% higher mean DHEAS levels (*P* < .001) than ND girls (n = 249). No differences were observed in glucose, insulin, homeostatic model assessment for insulin resistance, lipid profile, testosterone, SHBG, free androgen index, or 11-oxosteroid concentrations. HD girls exhibited slightly higher mean blood pressure but no increased prevalence of metabolic syndrome in crude or adjusted models (adjusted for birth weight SD score, age at menarche, and body mass index at ∼7 years). Among HD girls, DHEAS at 7 years was not correlated with 11-oxosteroids at 4YPM. Serum 11KA4 was inversely associated with glucose after adjustment (β −.02; 95% CI, −0.04 to −0.006).

**Conclusion:**

Elevated DHEAS at adrenarche persists after puberty without higher bioactive 11-oxosteroids or significant metabolic deterioration, suggesting attenuation of early adverse metabolic features by mid-adolescence.

## Introduction

Adrenarche denotes the maturation of the adrenal zona reticularis and its increased synthesis of 19-carbon (C19) steroids, particularly dehydroepiandrosterone and its sulfate (DHEAS). The increase in these steroids is apparent at ages 6 to 8 years [1]. Clinical manifestations of adrenarche include onset of axillary and/or pubic hair growth (pubarche), adult body odor, and mild acne, all of which indicate increased androgen activity. Premature adrenarche (PA) is defined biochemically by circulating DHEAS concentrations >40 μg/dL (1.08 μmol/L) by radioimmunoassay (HD) or >25 μg/dL by liquid chromatography-tandem mass spectrometry [2] before the age of 8 years in girls and 9 years in boys and is recognized clinically by the signs described here [1].

Recently, liquid chromatography-tandem mass spectrometry studies have demonstrated human adrenal production of potent 11-oxygenated C19 androgens. Among them, 11-ketotestosterone (11KT) is the dominant circulating bioactive androgen during normal and PA and is an important biomarker of adrenal hyperandrogenism [3].

Although PA has long considered to be a benign variant of development, recent studies have questioned this conclusion. Studies in girls with idiopathic PA from different ethnic backgrounds link this condition to an increased frequency of metabolic syndrome (MetS) at the time of presentation [4] and, in a subset of girls, to greater susceptibility to polycystic ovary syndrome [5]. However, controversy exists as to whether previously described associations between PA and obesity may depend on the ethnic background and prevalence of low birth weight in the study population.

Previous data published by our group have investigated the potential association between PA and pubertal development [6], ovarian hyperandrogenism [7], and metabolic dysfunction [8]. We have used a biochemical definition of adrenarche to identify premature adrenarche independent of clinical manifestations that may depend on ethnicity, steroid metabolism, sensitivity to androgens, or environmental factor [9-11]. In these girls, we observed at the age of adrenarche that all adiposity indicators (body mass index [BMI], waist circumference, percent fat, waist/height) were positively and similarly associated with DHEAS and that these associations were only partially related to IGF-I and leptin and not to insulin [12]. Throughout puberty up to 1-year postmenarche (1YPM), girls with high DHEAS (HD = DHEAS concentrations >75th centile of the general population) at age ∼7 years exhibited higher BMI. At 1YPM, they also showed ∼5 times greater prevalence of metabolic syndrome, and those with BMI >1 SD score (SDS) had a higher metabolic score and insulin levels than girls with similar BMI but normal DHEAS (ND) at age of adrenarche. This observation shows that HD at the age of adrenarche in girls is associated to a risk for metabolic syndrome at adolescence, especially in those who are overweight or obese [8].

We have hypothesized that the features observed at 1 year PM may persist later in puberty and therefore have planned a further analysis at 4 years postmenarche (4YPM). Additionally, because 11-oxygenated C19 androgens are the most potent adrenal androgens, we have sought to determine in this cohort whether the concentration of DHEAS and 11-oxygenated C19 androgens correlates at 4YPM and whether these adrenal steroids are associated with metabolic disturbances in late puberty.

## Subjects and methods

Girls participating in this study have been recruited through the Growth and Obesity Chilean Cohort Study, which recruited 602 girls in 2006 at the age of 3.0 to 4.9 years who were attending the Chilean National Nursery School Council Program in the southeastern area of Santiago, Chile. A comprehensive detail of this cohort can be found elsewhere [13]. Briefly, the inclusion criteria for participating in the Growth and Obesity Chilean Cohort Study were term singletons born in 2002 and 2003 with an appropriate birthweight for gestational age. At recruitment, we excluded children with physical, medical, mental, or endocrine diseases that may alter puberty.

The Ethics Review Board of the Institute of Nutrition and Food Technology of the University of Chile approved the study protocol (#028-2019). All parents or guardians of the children provided written informed consent, and the children gave their assent. These girls have been followed annually and pubertal development was assessed from age ∼6.7 years—by a single pediatric endocrinologist (V.M.)—with inspection and palpation of the breast according to the Tanner scale, and with puberty initiation defined as permanent breast stage Tanner ≥2 [6, 14]. Subsequently, secondary sex characteristics were evaluated every 6 months by a single-trained female dietitian, with supervision by the same pediatric endocrinologist (V.M.) and concordance between the dietitian and V.M. of 0.9 [14]. Age at menarche was self-reported, and telephone follow-up was performed every 6 months starting at breast Tanner Stage 4 (B4). After menarche an annual visit was scheduled until 4YPM for complete anthropometric measures and a fasting blood sample.

At age ∼7 years, the girls were classified into those with high fasting serum DHEAS levels (HD, >42.1 μg/dL [1.14 μmol/L], the 75th percentile of our population) or normal DHEAS (ND, ≤75th percentile) [12].

### Anthropometric measures

Weight, height, and waist circumference were determined using standard protocols [8]. Body composition was assessed using bioimpedance analysis with a TANITA Segmental Body Composition Analyzer (model BC-418). Subjects were instructed to be fasted and with voided urine. Blood pressure (BP) was measured with a digital sphygmomanometer, OMRON 705-IT, LUFKIN W606PM. Participants were seated with their arm on a desk and had rested at least 10 minutes before measurement. The BP measurement was performed 4 times. The first measure was discarded and the mean of the last 3 measures were used as estimates of systolic and diastolic BP. Hypertension was defined per the International Diabetes Federation criteria for metabolic syndrome [15].

### Circulating hormone and metabolite determinations

The day before clinic appointment mothers confirmed the absence of acute infection or fever in the children. A fasting early morning venous sample was collected. At age ∼6.7 years, serum DHEAS was determined by competitive specific binding radioimmunoassay (Diagnostic System Laboratories); the intra- and interassay coefficients of variation (CVs) were 3.5% and 5.1%, respectively. Thereafter, samples were obtained throughout puberty and at 4YPM. After 1YPM menarche samples were obtained in the early follicular phase (days 2-7 of the menstrual cycle) before 8:30 Am. For the 4YPM measurements, 73% of the fasting blood samples were obtained at this timing, whereas the remaining samples were obtained in late follicular phase. Serum glucose was measured by the enzymatic colorimetric method and expressed in mg/dL (mmol/L), and serum insulin by electrochemiluminescent immunoassay and expressed in μU/mL (pmol/L) with a detection sensitivity limit of 0.4 μU/mL (2.8 pmol/L) and an intra- and interassay CV of 1.6% and 2.2%, respectively. Serum lipid profiles (triglycerides [TGs], high-density lipoprotein cholesterol [HDL-C], low-density lipoprotein cholesterol, and total cholesterol) were determined by a dry analytic methodology (Vitros, Johnson & Johnson, Inc.). The measurements of serum SHBG (sensitivity = 0.5 nmol/L) was performed using an immunoradiometric assay (Izotop Laboratories, Budapest, Hungary). The intra- and interassay coefficients of variation for SHBG were 3.9% and 6.9%, respectively.

Concentrations of DHEAS, androstenedione, and testosterone were analyzed by liquid chromatography-mass spectrometry in a HPLC Agilent system (Santa Clara, CA, USA) 1260 coupled to an AB Sciex 3200 Quantum ultratriple quadrupole mass spectrometer (Foster City, CA, USA) as previously described [7]. The quantification of the 11-oxygenated androgens 11-hydroxyandrostenedione (11OHA4), 11-hydroxytestosterone (11OHT), 11-ketoandrostenedione (11KA), and 11KT was performed by liquid chromatography-tandem mass spectroscopy as described previously [1]. The sensitivities for 11OHA4, 11KA4, 11OHT, and 11KT were 0.010, 0.032, 0.010, and 0.032 ng/mL (33.1, 105.9, 33.1, and 105.9 pmol/L), respectively. The corresponding intra-assay CVs were 4.4%, 3.6%, 5.1%, and 3.9%, respectively, and the inter-assay CVs were 6.1%, 4.7%, 6.9%, and 5.2%, respectively [2].

Homeostatic model assessment for insulin resistance (HOMA-IR) was calculated as fasting serum glucose (mg/dL)/fasting serum insulin (mU/L)/405 (fasting glucose [nmol/L]/fasting insulin [mU/L])/22.5). Hyperglycemia was defined as fasting glucose concentration >5.55 mmol/L, hypertriglyceridemia as fasting levels ≥1.69 mmol/L, and low HDL-C as fasting HDL-C concentration <1.29 mmol/L. Dyslipidemia was defined either by lipid subfractions (TG level >1.69 mmol/L or HDL-C <1.29 mmol/L) or by calculated TG-to-HDL-C ratio (>5.0) [15].

Additionally, we used an additional definition as proxies for IR: MetS and its components based on the International Diabetes Federation [15] and MetS score derived by standardizing each component according to our sample distribution and then summing the following continuously distributed variables: waist circumference (WC), mean of systolic and diastolic BPs, fasting insulin, fasting glucose, and the TG-to-HDL-C ratio [16]. SHBG and testosterone were used to calculate the free androgen index (FAI) [17].

#### Anthropometrics computed indexes

The BMI for age, weight-for-age, height-for-age, and BMI-for-age SDS were calculated based on the World Health Organization 2007 growth references [18]. We defined overweight and obesity as BMI SDS ≥1 to <2 and ≥2, respectively. Central obesity was defined by WC ≥ 90th percentile, based on the NHANES II 90th percentile for the Hispanic population [19].

#### Statistical analysis

Girls were placed into the HD or ND group based on the sample obtained in 2009 at ∼7 years of age. Complete pubertal data have been reported previously [8]. Descriptive analyses (mean, SDS, and percentage) of anthropometric, hormonal, and sexual maturation data were performed for the HD and ND groups. Statistical differences between the groups (HD vs ND) were assessed by χ^2^ and Student *t* test, as appropriate, and the Mann-Whitney test was used to compare differences between median hormonal levels. Multiple linear regression models were used to assess the relationship among DHEAS at age ∼7 years, metabolic syndrome, and each of its components independently at 4YPM, with adjustment for chronological age at DHEAS sampling, BMI SDS at age of DHEAS sampling, and birth weight. Using Pearson's correlation coefficient, we next analyzed the correlation at 4YPM among 11-oxygenated C19 androgens. Subsequently, we performed multiple linear regression models (as previously described) to assess the relationship between each of these reticularis-derived steroid products and the metabolic syndrome at 4YPM. Analysis was carried out in STATA version 16.0, and the results were considered significant at a *P* < .05.

## Results

### Subjects

From the 504 girls who had DHEAS measured at age ∼7 years, data from 345 were available for the current study (68%), whereas data from 159 girls were not available. The 159 girls not available at 4YPM had—at age ∼7 years—similar age, height, BMI, WC, waist/hip ratio, percent obesity and overweight, birth weight SDS, and weight change between birth and 2 years compared to the girls who were evaluated at 4YPM (N = 345). Nevertheless, those unavailable from the full 4YPM cohort had slightly higher percent body fat (25.4 ± 4.3 vs 24.5 ± 4.2, *P* < .05) than those included in the current analysis (data not shown).

At 4YPM, 96 girls were classified as HD (27.8%) and 249 as ND (72.2%) by DHEAS concentration at age ∼7 years. Mean ± SD age was 16 ± 0.8 years and birthweight, gestational age, and weight SDS change from birth to age 2 years were similar between HD and ND groups. In addition, mean heights were within the normal ranges (± 2 SDS) and BMIs within the eutrophic range.

At 4YPM, the girls with HD were slightly younger (*P* < .01) and had higher BMI SDS (*P* < .05), higher waist/height ratio (*P* < .05), higher waist/hip ratio (*P* < .05), and higher percentages of obesity and overweight (*P* < .05) compared to the girls with ND (Table 1).

**Table 1:** Anthropometric characteristics at 4YPM stratified by HD and ND at age 7 years

	HD (n = 96)	ND (n = 249)	*P* value
	Mean ± SD	Mean ± SD	
Age at 4 YPM (y)	15.8 ± 0.8	16.1 ± 0.8	.007
Weight (kg)	63.5 ± 11.8	61.6 ± 12.8	.213
Height (cm)	158.4 ± 5.9	159.7 ± 5.7	.062
Height SDS	−0.54 ± 0.9	−0.39 ± 0.8	.118
BMI SDS	1.1 ± 1.0	0.8 ± 1.0	.013
WC (cm)	80.1 ± 10.3	77.9 ± 10.7	.072
Weight SDS change 0-2 years > 0.67 SDS (n, %)	24 (32.0)	70 (36.1)	.529
Nutritional status			
Normal, n (%)	40 (41.7)	146 (58.9)	.015
Overweight, n (%)	36 (37.5)	68 (27.4)	
Obesity, n (%)	20 (20.8)	34 (13.7)	
Central obesity, n (%)	7 (7.29)	14 (5.6)	.561
WC/height ratio	0.51 ± 0.06	0.49 ± 0.07	.015
WC/hip ratio	0.80 ± 0.06	0.79 ± 0.06	.014
Body fat percentage %	33.4 ± 7.0	32.5 ± 6.9	.330
Systolic blood pressure (mm Hg)	107.2 ± 0.95	104.5 ± 0.56	.014
Diastolic blood pressure (mm Hg)	62.1 ± 0.72	60.3 ± 6.7	.025

Abbreviations: 4YPM, 4 years postmenarche; HD, high DHEAS (>75th percentile); ND, normal DHEAS (≤75th percentile) at age 7 years; WC, waist circumference.

### Hormonal profile

Four years after menarche, mean DHEAS concentration in the HD girls was ∼47% higher than in the ND girls. There was no difference in the proportion of participants with samples obtained in the early follicular phase (75% in ND and 70% in HD girls, *P* = .311) . Furthermore, there were no differences in the DHEAS concentrations obtained at early follicular vs late follicular phase within participants with HD or ND groups (*P* = .2). Nevertheless, serum glucose and insulin, HOMA-IR, total cholesterol and subfractions, triglycerides, testosterone, SHBG, FAI, and concentrations of all 4 11-oxygenated C19 steroids, did not differ compared to girls with ND (Table 2).

**Table 2:** Metabolic and hormonal profile at 4YPM stratified by DHEAS concentrations at age 7

	HD (n = 96)	ND (n = 249)	*P* value
	Mean ± SD	Mean ± SD	
Serum glucose (mmol/L)	4.53 ± 0.41	4.47 ± 0.35	.21
Serum insulin (pmol/L)	57.6 ± 20.8	59.7 ± 21.5	.53
HOMA-IR	1.69 ± 0.7	1.70 ± 6.4	.84
Triglycerides (nmol/L)	1.11 ± 0.55	1.10 ± 0.54	.69
HDL-C (mmol/L)	1.34 ± 0.29	1.32 ± 0.27	.65
LDL-C (mmol/L)	1.63 ± 0.42	1.71 ± 0.49	.21
TG/HDL-C	1.99 ± 1.00	1.96 ± 0.90	.84
C/TG	1.57 ± 0.60	1.59 ± 0.50	.74
DHEAS (µmol/L)	5.37 ± 1.83	3.66 ± 1.49	.0001
Testosterone (nmol/L)	0.94 ± 0.35	0.90 ± 0.35	.90
Androstenedione (nmol/L)	4.71 ± 1.81	4.40 ± 1.82	.14
SHBG (nmol/L)	51.6 ± 39.1	57.6 ± 40.7	.23
FAI	2.7 ± 2.7	2.50 ± 2.8	.52
11OH A (pmol/L)	562.7 ± 231.7	560.5 ± 264.8	.89
11OH T (pmol/L)	459.2 ± 229.6	426.4 ± 196.8	.14
11 K A (pmol/L)	832.5 ± 399.8	829.7 ± 395.7	.99
11 K T (pmol/L)	397.2 ± 231.7	364.1 ± 198.6	.35

Abbreviations: 4YPM, 4 years postmenarche; C, cholesterol; FAI, free androgen index; HD, high DHEAS (>75th percentile); HDL, high-density lipoprotein; HOMA-IR, homeostatic model assessment for insulin resistance; LDL, low-density lipoprotein; ND, normal DHEAS (≤75th percentile) at age 7 years; TG, triglyceride.

**Figure 1:**
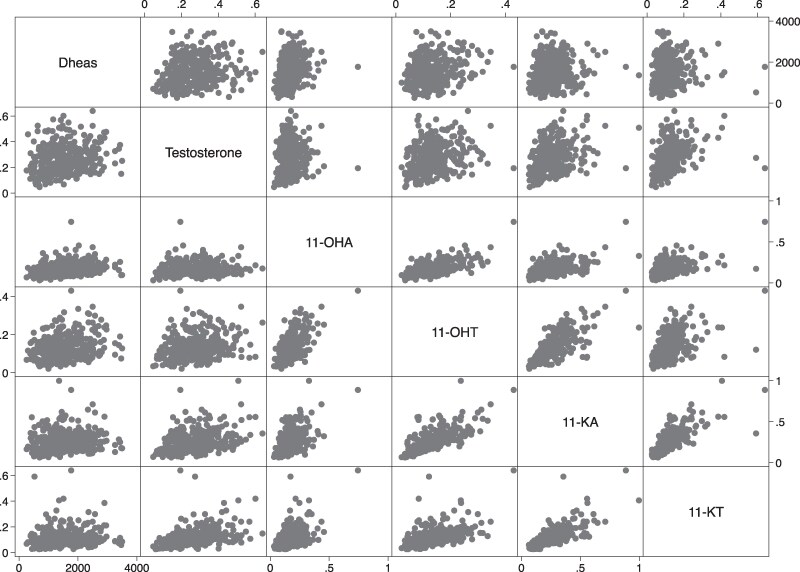
Correlation matrix plot between DHEAS, testosterone, and 11-oxygenated C19 androgens.

In combined HD and ND groups, DHEAS concentrations at 4YPM correlated significantly with concentrations of testosterone (*r* = 0.42, *P* < .0001), 11-KA4 (*r* = 0.25, *P* < .0001), 11OHA4 (*r* = 0.26, *P* < .0001), and 11OHT (*r* = 0.12, *P* < .05). Furthermore, testosterone concentrations correlated with all 4 11-oxygenated C19 steroids: 11KA4 (*r* = 0.13, *P* < .05), 11OHA4 (*r* = 0.29, *P* < .0001), 11KT (*r* = 0.36, *P* < .001), and 11OHT (*r* = 0.40, *P* < .001). A correlation matrix plot between DHEAS, testosterone, and 11-oxygenated C19 androgens is shown in Fig. 1.

### Metabolic syndrome and metabolic score

At 4YPM, only 12 girls had a diagnosis of MetS (7 were ND and 5 were HD); therefore, because of sample size we analyzed only the relation between DHEAS group and metabolic score. HD was not associated with a higher metabolic score in the crude and adjusted models (covariates: birth weight SDS, age at menarche, and BMI at age ∼7). However, HD was associated with higher mean BP (Table 3). Among the 11-oxygenated androgens, only 11KT concentration was positively associated with a higher mean BP in the adjusted model (Table 4). 11KA and 11KT were inversely associated with serum glucose in the crude model, but only the former remained significant after adjustment (Table 4). Also, 11KA and 11KT were inversely associated with metabolic score and WC in the crude model.

**Table 3:** Linear regression models to assess the association between biochemical adrenarche (HD vs ND) at age 7 years and metabolic score and its standardized components independently at 4YPM

	β	95% CI	β*^a^*	95% CI
Metabolic score SDS	.49	−0.1 to 1.1	.2	−0.2 to 0.7
WC SDS	.2	−0.02 to 0.4	.006	−0.09 to 0.1
Serum glucose SDS	.14	−0.08 to 0.35	.9	−0.1 to 0.3
Serum insulin SDS	−.05	−0.2 to 0.1	−.07	−0.2 to 0.08
Serum TG/HDL-SDS	.02	−0.2 to 0.2	−.04	−0.2 to 0.2
Blood pressure SDS	.3	0.08-0.5	.2	0.03-0.4

Abbreviations: 4YPM, 4 years postmenarche; blood pressure, mean of systolic and diastolic values; HD, high DHEAS (>75th percentile), ND, normal DHEAS (≤75th percentile) at age 7 years; WC, waist circumference.

^*a*^Adjusted by birthweight SD score, age at menarche, and body mass index SD score at age 7 years.

**Table 4:** Linear regression models to evaluate the association between 11-oxogenic androgens and DHEAS concentrations and metabolic score and its standardized components independently at 4YPM

	11-OH A				11-OH T				11-KA				11-KT				dheas			
	β	95% CI	β	95% CI	β	95% CI	β	95% CI	β	95% CI	β	95% CI	β	95% CI	β	95% CI	β	95% CI	β	95% CI
Metabolic score SDS	−.001	−0.004; 0.003	.002	−0.002; 0.006	.0007	−0.002; 0.003	.0006	−0.003; 0.004	−.01	−0.017; −0.006	−.003	−0.010; 0.004	−.005	−0.008; −0.001	−.0007	−0.005; 0.003	−2.67	−32.2; 26.8	−18.9	−56.7; 18.9
Waist Circum SDS	−.005	−0.014; 0.004	.015	−0.006; 0.036	.005	−0.003; 0.012	.019	0.002; 0.036	−.03	−0.05; −0.02	.02	−0.02; 0.05	−.013	−0.021; −0.005	.013	−0.006; 0.033	−1.4	−91.5; 78-8	−92.7	−274.8;89.4
Glycemia SDS	.003	−0.007; 0.012	.004	−0.005; 0.014	−.004	−0.012; 0.003	−.004	−0.012; 0.003	−.03	−0.046; −0.015	−.02	−0037; −0.006	−.01	−0.019; −0.002	−.008	−0.0169; 0.0008	17.4	−65.0; 99.8	2.1	−82.3; 86.4
Insulin SDS	−.0004	−0.013; 0.012	−.0007	−0.014; 0.012	−.001	−0.012; 0.009	−.001	−0.012; 0.009	−.005	−0.026; 0.016	−.002	−0.023; 0.018	−.007	−0.019; 0.005	−.006	−0.018; 0.005	7.6	−103.4; 118.6	−10.2	−120.6; 100.2
TG/HDL-SDS	.003	−0.006; 0.013	.006	−0.004; 0.017	.002	−0.006; 0.009	.002	−0.006; 0.010	−.015	−0.0317; 0.0004	−.004	−0.02; 0.01	−.01	−0.019; −0.001	−.005	−0.014; 0.004	−56.4	−141.0; 28.2	−79.6	−167.1; 7.9
Blood pressure SDS	−.006	−0.015; 0.003	−.004	−0.013; 0.006	.003	−0.004; 0.010	.0025	−0.005; 0.010	−.005	−0.02; 0.01	.006	−0.01; 0.02	.002	−0.006; 0.010	.01	0.001; 0.018	10.7	−66.5; 87.9	3.0	−78.4; 84.4

Furthermore, there was no association between HD at age ∼7 years and the 11-oxygenated C19 steroid concentrations at 4YPM either as continuous variables (Table 5) or as categorical variables (ie, 11-oxygenated C19 steroids ≤75th percentile vs >75th percentile [data not shown]).

**Table 5:** Linear regression models to assess the association between the concentrations of DHEAS at ∼7 years in the HD group and those of each 11-oxygenated androgen at 4 yr postmenarche

	β	95% CI	β*^a^*	95% CI
11OHA	−.001	−0.02 to 0.02	−.01	−0.03 to 0.01
11OHT	.01	−0.003 to 0.03	.01	−0.01 to 0.02
11KA	−.0002	−0.03 to 0.03	.005	−0.02 to 0.03
11KT	.01	−0.01 to 0.02	.01	−0.01 to 0.02

Abbreviation: HD, high DHEAS (>75th percentile).

^*a*^Adjusted by birth weight SD socre, age at menarche, and body mass index SD score at age 7 years.

## Discussion

The current study examined the hypotheses that girls with high DHEAS levels at age 7 years would have an increased prevalence of MetS and its individual components at 4YPM, and that these features would be more closely associated with the adrenal 11-oxysteroids than with DHEAS concentrations. Apart from higher BP, BMI SDS, waist/height and waist/hip ratios, more overweight/obese proportion, the unfavorable metabolic and androgenic signature (ie, glucose, HOMA-IR, lipid profile, testosterone, SHBG, FAI, and concentrations of all 4 11-oxygenated C19 steroids) in girls with HD at age 7 years appeared to diminish at 4YPM despite the persistence of higher DHEAS levels after puberty completion.

This observation was unexpected but highlights that the associations found earlier during adrenarche and early puberty were not necessarily causal relationships. Moreover, our results seems consistent with the finding that the higher DHEAS levels at 4YPM in the HD group were, also unexpectedly, not accompanied by increased concentrations of the more bioactive 11-oxygenated adrenal androgens. Our study provides no mechanistic insight for this apparent dissociation of the delta 5 and delta 4 pathways to adrenal androgens and 11-oxoandrogens. However, potential explanations include age-related changes in the steroid sulfatase, the adrenal 3-beta-hydroxysteroid dehydrogenase, or the zona reticularis vs fasciculata contributions to 11-oxyandrogens (via cortisol metabolism to 11-OHA). Additionally, DHEAS is tightly bound to albumin and relatively invariant, whereas the 11-oxyandrogens are weakly bound to SHBG because of their 11-oxo group and thus subject to rapid degradation and larger circadian and stress-related changes. For the overall cohort, however, statistically significant correlations were observed between the levels of DHEAS and testosterone, 11OHA, 11KA, and 11OHT.

The measured levels of 11-oxosteroids in our HD Chilean cohort were similar to those reported in overweight/obese Tanner 5 girls [20]. Additionally, our results for 11-OHT and 11-KT during adrenarche [2] were like those reported by Rege and colleagues [3]. However, the level of 11-KT in our HD cohort was only about one third of the level seen in women with polycystic ovary syndrome [21].

Interestingly, at 4YPM, our girls with HD persisted in having higher BMI SDS, although the percent of girls with overweight and obesity decreased compared to 1YPM in both the HD and ND groups. Additionally, the girls with HD did have a numerically greater frequency of metabolic syndrome (5/96 [5.2%] vs 7/294 [2.8%]) but this difference did not achieve statistical significance. Also, the greater serum glucose observed during early puberty waned. Similarly, higher androstenedione, testosterone, and FAI observed during earlier stages of puberty through 1YPM did not persist. Most of these results are in line with observations from other studies in girls with premature adrenarche [21-23].

The main limitation of this study relates to the decision, for statistical reasons, to analyze the hormonal and metabolic differences between the upper quartile vs the lower 3 quartiles of DHEAS concentration. Specifically, an absence of differences between the upper 25th percentiles and lower 75th percentiles should not be interpreted as indicating what would be observed, for example, in a comparison between the upper 2.5th percentiles vs the lower 97.5th percentiles. Also, because our subjects are still young, these data should not be interpreted as foreclosing the possibility of later metabolic or reproductive complications related to their early life increased BMI, DHEAS, and 11-oxyandrogens. The main strengths of the study are its prospective, long-term, longitudinal design with consistent anthropometric, metabolic, and hormonal measures throughout.

We conclude that girls with high DHEAS at age 7 years continue to have high DHEAS, but not of the more potent 11-oxygenated adrenal androgens, after completion of puberty at 4 years postmenarche. Apart from higher BP and increased adiposity, however, the unfavorable metabolic signature at adrenarche appeared to ameliorate at 4 years postmenarche.

## Data Availability

Some or all datasets generated during and/or analyzed during the current study are not publicly available but are available from the corresponding author on reasonable request.

## Abbreviations

1YPM: 1 year postmenarche
11KT: 11-ketotestosterone
11OHA4: 11-hydroxyandrostenedione
11OHT: 11-hydroxytestosterone
4YPM: 4 years postmenarche
BMI: body mass index
BP: blood pressure
C19: 19 carbon
CV: coefficient of variation
DHEAS: dehydroepiandrosterone sulfate
FAI: free androgen index
HD: high dehydroepiandrosterone sulfate
HDL-C: high-density lipoprotein cholesterol
HOMA-IR: homeostatic model assessment for insulin resistance
MetS: metabolic syndrome
ND: normal dehydroepiandrosterone sulfate
PA: premature adrenarche
SDS: SD score
TG: triglyceride
WC: waist circumference
